# Tailored Diluent for Nanostructure and Viscosity Control in an Ionic Liquid

**DOI:** 10.1021/acs.jpcb.6c03434

**Published:** 2026-07-27

**Authors:** Shurui Miao, Yilang Chen, Frederik Philippi, Thomas Zinn, Kuno Schwärzer, Jia-Fei Poon, Lauren Matthews, Margarida Costa Gomes

**Affiliations:** † Physical and Theoretical Chemistry Laboratory, Department of Chemistry, 6396 University of Oxford, Oxford OX1 2JD, U.K.; ‡ Laboratoire de Chimie de l’ENS Lyon, CNRS and Université de Lyon, Lyon 69007, France; § Diamond Light Source, Diamond Light Source Ltd., Harwell Campus, Didcot OX11 0DE, U.K.; ∥ Jülich Centre for Neutron Science (JCNS-1), Forschungszentrum Jülich GmbH, Wilhelm-Johnen-Straße, Jülich 52428, Germany; ⊥ 338799 European Spallation Source, Lund, Box 176 SE-221 00, Sweden; # ISIS Neutron and Muon Source, Rutherford Appleton Laboratory, Harwell Campus, Didcot OX11 0QX, U.K.

## Abstract

Ionic liquids (ILs) offer tunable physicochemical properties but are often limited by their high viscosities. This is commonly addressed by introducing low-viscosity diluents, which increase molecular complexity and frequently lead to nonlinear viscosity–composition relationships. Here, we designed a molecular diluent that mimics the parent IL. We demonstrate that this approach enables viscosity tuning over 3 orders of magnitude while preserving the self-assembled nanostructure of the neat IL across all compositions. This work establishes a new strategy for designing IL–diluent systems and provides a model platform to investigate the role of Coulombic interactions in governing the local structure and macroscopic properties.

## Graphical abstract

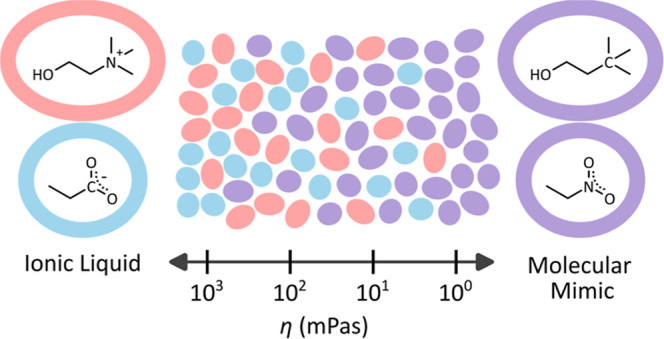

## Introduction

Ionic liquids (ILs) are low-melting salts that are often referred to as designer solvents due to the synthetic flexibility in formulating these low-melting-point salts. They exhibit a range of desirable physicochemical properties, including relatively high thermal and electrochemical stability, negligible vapor pressure, and high ionic conductivity. These bulk properties can also be tuned through the choice of constituent ions, enabling a task-specific design and optimization. As a result, ionic liquids have shown promise in a variety of applications, including biomass processing,^1,2^ chemical separations and recycling,^3,4^ and as novel electrolytes in energy storage technologies, such as batteries and supercapacitors.^5,6^ However, the practical applicability of many IL-based functional fluids is limited by their high viscosity compared with conventional molecular solvents.^7^ This challenge is often addressed by the introduction of a low-viscosity molecular diluent. To date, the design strategy of such IL–diluent mixtures has often relied on trial-and-error approaches, which greatly limits our ability to effectively explore the vast design space available for these systems.

In ILs, many of their properties arise from the presence of interpenetrating ionic networks sustained by a delicate balance of local intermolecular interactions and Coulombic forces. The origin of these nanostructures is now reasonably well understood, and it is evident that they play an important role in translating molecular features into macroscopic properties.^8,9^ However, when a diluent is introduced to regulate the viscosity, it often modifies these nanostructures and compromises the desired properties of the pure IL.^10^ As a result, a conundrum arises: improving the handleability of IL-based mixtures often requires sacrificing the functional performance. Therefore, an ideal diluent should preserve the IL nanostructure and chemical functionalities while providing a handle for the bulk viscosity.

In this study, we have designed an IL (choline propionate) and its corresponding molecular mimic (MM).^7,11^ The MM is a 1:1 (molar ratio) mixture of 3,3-dimethylbutan-1-ol (DMB) and nitroethane (NE), neutral analogues of the cation and anion of the IL. The ion mimics are formally constructed by switching the positions of the carbon and the nitrogen atom (Figure 1). The MM is therefore isoelectronic, isostructural, and isobaric to the IL and is expected to have similar intermolecular interactions as the parent IL, with the only exception of Coulomb interactions (ab initio calculations of molecular volume in Supporting Information). We hypothesize that the MM should be an ideal diluent for the IL. In a mixture of IL and its MM, the charge density is lowered while maintaining all other interspecies interactions to the extent possible. Despite their similarities, the bulk viscosity of the MM is almost 3 orders of magnitude smaller than that of the IL. By studying the effect of the molecular diluent on liquid nanostructure using small-angle X-ray scattering, we test our hypothesis and create a model system to directly study the effect of a charged network on the structure of ionic liquids and concentrated electrolytes in general.

**Figure 1:**
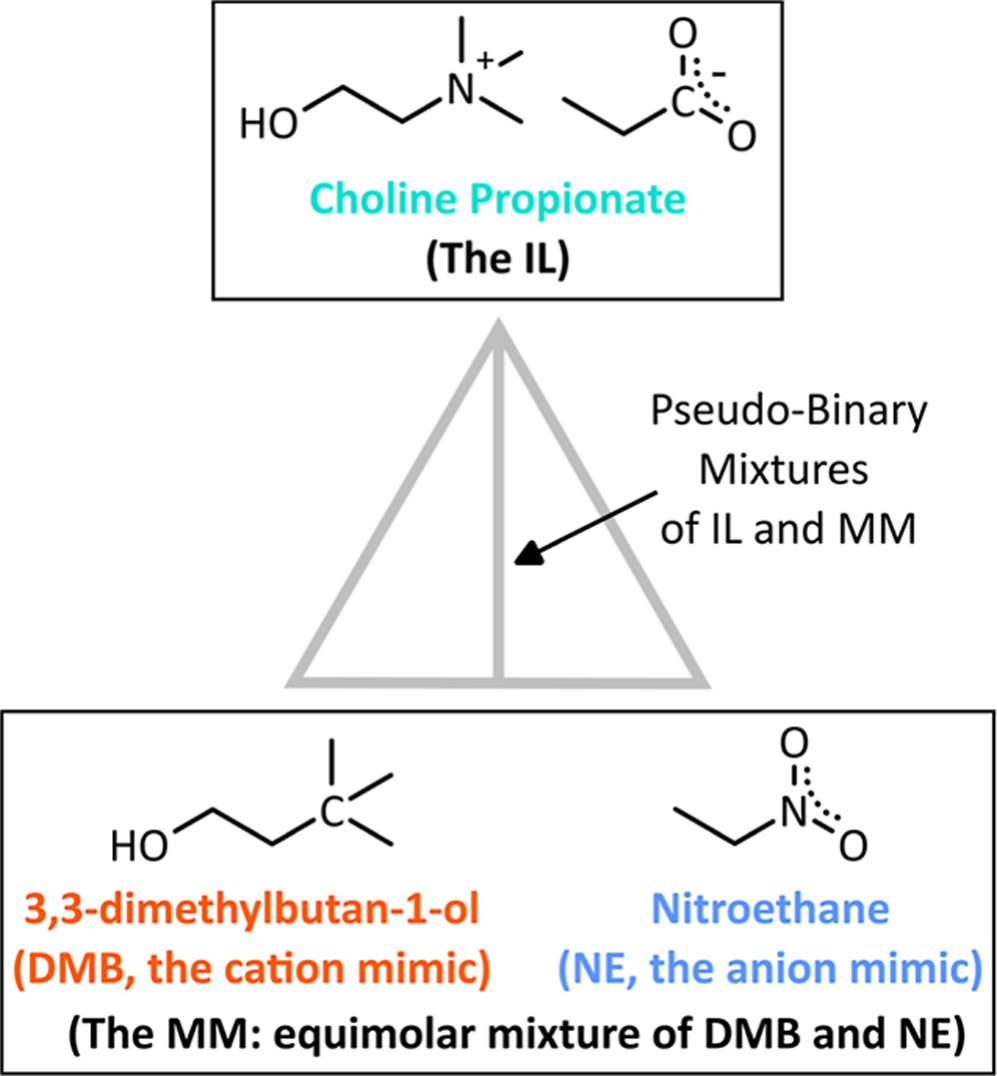
Molecular structures of the IL (choline propionate), the cation mimic (3,3-dimethylbutan-1-ol), and the anion mimic (nitroethane). We will study the three binary mixtures and the ternary mixture along the gray lines. Compositions along the vertical gray line are treated as a pseudobinary mixture of the IL and MM, since the MM is defined by a fixed 1:1 NE/DMB ratio.

## Materials and Methods

### The Ionic Liquid and Its Molecular Mimic

Choline hydroxide (aqueous solution, ∼15 wt %, Iolitec, >97% pure), propionic acid (≥99.5%, Sigma-Aldrich), nitroethane (≥98.0%, Sigma-Aldrich), and 3,3-dimethyl-1-butanol (98%, Sigma-Aldrich) were purchased. Nitroethane and 3,3-dimethyl-1-butanol were dried over fresh, activated (1.2 μbar end vacuum, 200 °C, overnight) molecular sieves (Sigma-Aldrich, 3 Å) for several days and then passed through a 100 nm Nylon syringe filter.

To make the ionic liquid (choline propionate), propionic acid was neutralized by choline hydroxide. The reaction mixture was rapidly stirred and cooled in an ice bath. pH was monitored using a calibrated pH meter (HI5221, HANNA Instrument); the end point was determined by a sudden jump in pH, and it was adjusted to 7.0. Water was removed on a rotary evaporator (30 mbar, 40 °C), followed by drying in an oil pump vacuum for 1 week with stirring at 1.2 μbar end vacuum. Water content was determined by coulometric Karl Fischer titration (HI934, HANNA Instrument) to be ∼2500 ppm.

Deuterated materials for small-angle neutron scattering were provided by Dr Kuno Schwärzer and Dr Jia-Fei Poon. Detailed procedures for the synthesis of choline hydroxide-d_13_, nitroethane-d_5_, and 3,3-dimethylbutan-1-ol-d_14_ are outlined in Supporting Information. Propionic acid-d_4_ (98%, Cambridge Isotope) was purchased and used as received. Preparation of the deuterated ILs is identical to the procedure described above, and D_2_O (99.9%, Sigma-Aldrich) was used as the solvent.

### Small Angle X-ray Scattering

SAXS patterns were recorded at the Diamond Light Source using the LabSAXS instrument. LabSAXS is a Xeuss 3.0 SAXS instrument (Xenocs, France) equipped with a liquid gallium and indium alloy MetalJet X-ray source (Excillum, Sweden, λ = 0.134 nm) operating at 70 kV with two sets of motorized scatter-less slits for beam collimation and an Eiger2 R 1 M pixel detector. SAXS patterns were recorded from *q* = 0.35 nm^–1^ to *q* = 9.35 nm^–1^, for which *q* = (4π sin θ)/λ is the length of the scattering vector, and θ is one-half of the scattering angle. Quartz capillaries of 1.5 mm diameter were used as sample holders, and patterns were recorded and averaged over 45 min. Data were reduced (normalized, integrated, and averaged) using standard routines within the DAWN software package. All samples were measured at 30, 50, and 70 °C; the full data set at 30 °C is shown below. The complete data set at all 3 temperatures can be accessed via the Oxford University Research Archive.

### Small-Angle Neutron Scattering

SANS was measured with deuterated chemicals to confirm no large aggregates or critical behaviors are present in IL–MM mixtures. SANS measurements were performed on the Loq instrument at the ISIS Neutron and Muon Source (STFC, Rutherford Appleton Laboratory, Didcot, UK). A *q*-range of 0.007–0.23 Å^–1^ was achieved using a 4 m configuration and neutrons with wavelengths of 2–10 Å. The raw scattering data were corrected for detector efficiency and sample transmission and were then normalized to an absolute intensity scale using Mantid. The data then underwent azimuthal averaging to obtain the 1D profiles, and the corresponding normalized intensity of an empty cuvette was subtracted from each data set (Figure S4).

### Density and Viscosity Measurements

Density and viscosity were measured on a combined densitometer/viscometer (SVM 3001, Anton Paar). Each sample was prepared and filled into a syringe in a glovebox. The syringe was taken out of the glovebox, immediately transferring and measuring the sample to minimize exposure to air and moisture. The experimental data are summarized in Table S1.

### NMR Diffusometry

The diffusion coefficients in the ionic liquid and molecular mimic differ by several orders of magnitude, in line with the viscosities, see Table S3. Hence, it was necessary to spread the measurements over two different spectrometers. A Bruker Avance III 400 MHz spectrometer equipped with a 5 mm CryoProbe Prodigy probe was used for low-viscosity samples, indicated in blue in Table S3. Samples with high viscosity were measured on a Bruker Avance III 400 MHz spectrometer equipped with a BBIDIff probe, indicated in red in Table S3. Some samples could be measured on both machines, indicated in green in Table S3. In our experience, the experimental uncertainty of PFGSTE diffusometry is around 10%; consistent with this, the average deviation between the two probe heads was approximately 6%.

## Results and Discussion

Self-assembly of amphiphilic liquids into nanostructures is well documented for many systems ranging from *n*-alkanols to ILs and their mixtures.^8,12–14^ Scattering experiments and simulation studies have identified multiple characteristic correlation lengths in nanostructured liquids. From short to long, they are attributed to the nearest neighbor, charge–charge, and polar–apolar separations.^15–17^ The nearest neighbor correlation is always present in liquids, while the charge–charge and polar–apolar correlations can only emerge when the constituent species are charged and/or amphiphilic.^17–19^ The scattering peak that corresponds to the polar–apolar correlation is often referred to as the prepeak and exhibits anti-Debye–Waller behavior.^15,20^ Small-angle X-ray scattering (SAXS) can effectively measure molecular correlation in this regime and is often employed to study self-assembly phenomena in liquids.

The small-angle X-ray scattering profiles of the three neat liquids and the MM are shown in Figure 2. Consistent with the literature, an IL prepeak is observed at a wavevector (*q*) ∼5 nm^–1^. This corresponds to a correlation length of around 1.2 nm, significantly greater than the nearest-neighbor correlation length in similar ILs at ∼0.5 nm.^20^ Interestingly, we observed a scattering feature resembling this prepeak in neat DMB and the MM on the same length scale. DMB contains a hydrocarbon tail and a hydroxyl group that engages in hydrogen bonding, enabling the formation of a liquid nanostructure similar to butan-1-ol.^12^ In contrast, the NE has a delocalized nitro group and a shorter hydrocarbon tail, limiting its ability to undergo amphiphilic self-assembly. Therefore, the scattering curve of NE is monotonically increasing toward its nearest neighbor peak just beyond our accessible *q*-range. When the DMB is mixed with 1 equiv of NE to form the MM, the prepeak-like feature in DMB is sustained but becomes broader, signaling the domains are more disordered and have a wider range of size distributions. While the MM is isoelectronic and isostructural with the IL, the lack of Coulombic interaction removes long-range electrostatic constraints on self-assembly, yielding more disordered correlations.^7^ For liquids and solutions with prepeak or prepeak-like features, the Teubner–Strey fit is often applied to extract the correlation length and the periodicity.^14,20^ However, due to constraints by the accessible *q*-range, we cannot reliably perform the Teubner–Strey fit to the entire data set; hence, the following discussion will focus on peak position and width extracted by a background-independent Lorentzian fit (details in Supporting Information).

**Figure 2:**
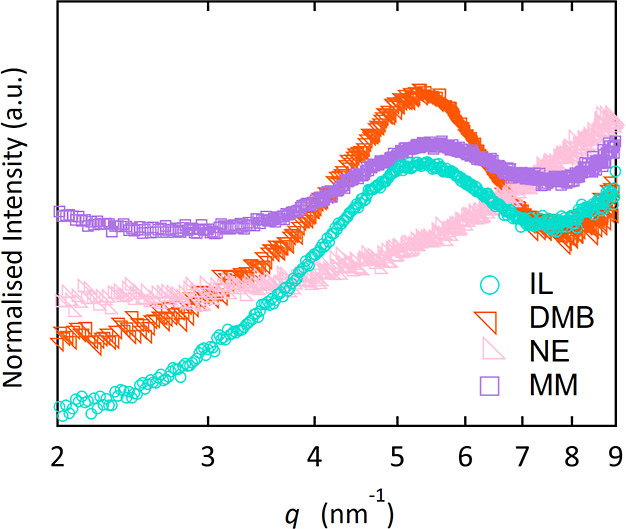
SAXS curves of the three neat substances and the molecular mimics (MM, equimolar mixture of DMB and NE). The peak around ∼5 nm^–1^ is the prepeak-like feature and corresponds to a correlation length longer than the nearest-neighbor separation.

Since DMB exhibits bulk self-assembly, we expected the IL–DMB mixtures to sustain the intermediate-range liquid structure at all compositions. This is confirmed by SAXS curves shown in Figure 3A, where a prepeak-like scattering feature is always present in all IL–DMB mixtures. Nevertheless, the peak position and its full width at half-maximum (FWHM) demonstrate a continuous evolution. At an IL mole fraction (χ_IL_) of 0.4, we observed a minimum in the peak position, which corresponds to a length scale that is significantly larger than the prepeak-like features in both the neat IL and neat DMB. A minimum in fwhm is also observed around this composition, indicating the underlying structural feature is also more uniform in size relative to the two components. Therefore, the liquid structure of IL–DMB mixtures is likely not a simple swelling of the inherent liquid structure of either the neat IL or the neat DMB. Such swelling should lead to continuous broadening of the IL prepeak with increasing diluent content.^20^ Instead, the IL–DMB mixtures likely undergo local demixing, forming interpenetrating and well-defined channels of charged and uncharged species. This is consistent with a previous study of choline ILs mixed with alkanols, where distinct bicontinuous networks of charged and neutral species were reported.^21^

**Figure 3:**
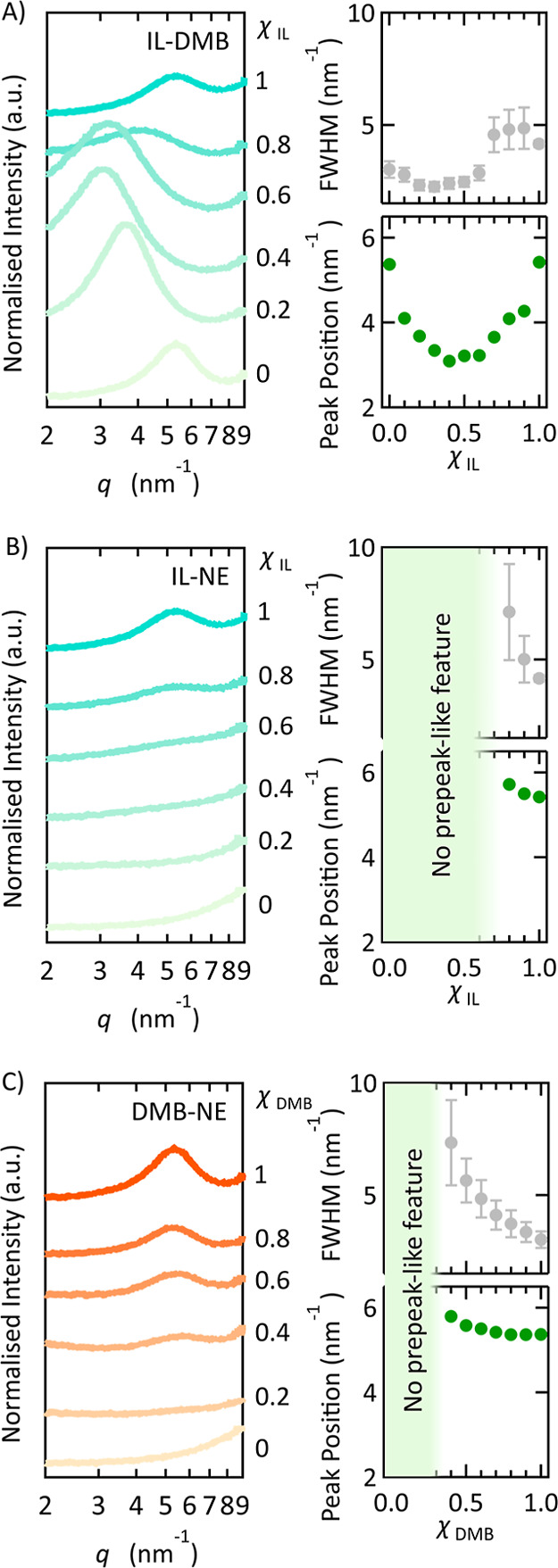
SAXS curves and fits of the three binary mixtures: (A) IL–DMB, (B) IL–NE, and (C) DMB–NE. Only representative curves are shown; the full data set is in Supporting Information. A small set of samples were also studied using small-angle neutron scattering to confirm the absence of larger aggregates (Supporting Information).

We also noticed that NE is surprisingly effective at disrupting the inherent IL nanostructure in IL–NE mixtures. Figure 3B shows that the IL prepeak becomes unidentifiable with 20 mol % of NE (∼10 wt %). With increasing NE content, the IL prepeak remains at the same position but becomes broader until it eventually completely disappears. This is consistent with swelling of the IL nanostructure where diluent is incorporated into the existing charged/polar network.^22^ The ability of NE to rupture inherent liquid structure is also evident in the DMB–NE mixtures in Figure 3C. NE is only a hydrogen bond acceptor; it will compete with DMB as acceptors but cannot sustain a continuous network. As a result, with increasing mole fraction of NE, it will disrupt the inherent alkanol clustering of DMB. The prepeak-like feature remained at the same *q*-range throughout the dilution but became broader and eventually undetectable once the mole fraction of NE reached 0.6 (i.e., χ_NE_ ≥ 0.6). This is consistent with the IL–NE mixtures where the IL nanostructure was disrupted by the NE and became undetectable once χ_NE_ ≥ 0.3. While the two mole fractions are quite different, the IL is composed of equal molar amounts of cation and anion; therefore, the underlying cation (or DMB) to (anion + NE) mole ratio is always near 1:2 when the nanostructure breaks down. This confirms that the propionate anion and neutral NE play almost identical roles in the observed liquid nanostructure.

The MM consisted of NE–DMB in a 1:1 mol ratio and was used to study the pseudobinary mixture of IL–MM (i.e., the fixed NE/DMB ratio of 1:1 reduces the composition to a single IL–MM axis). This enables us to continuously tune the charge density of our mixture while maintaining other molecular features of the neat IL to the extent possible (i.e., molecular size, weight, and all noncharged functional groups). A distinct prepeak-like feature is observed over the entire composition range of IL–MM mixtures. Like IL–DMB mixtures, it evolves in both the peak position and width with extrema around the 1:1 mol ratio (Figure 4). The correlation length of the IL–MM mixture reaches up to around 1.9 nm at the equimolar composition, significantly larger than in the neat IL and MM. This behavior closely resembles the IL–DMB mixture and therefore raises the question of whether the observed structure in IL–MM is due to IL–DMB segregation or IL–MM segregation. While the atom–atom pair correlation functions cannot be obtained from SAXS alone, which hindered us from resolving the molecular origin and differentiating the aforementioned segregations, we clearly showed that both IL–DMB and IL–MM mixtures exhibit intermediate-range structures in the bulk across the entire composition range.

**Figure 4:**
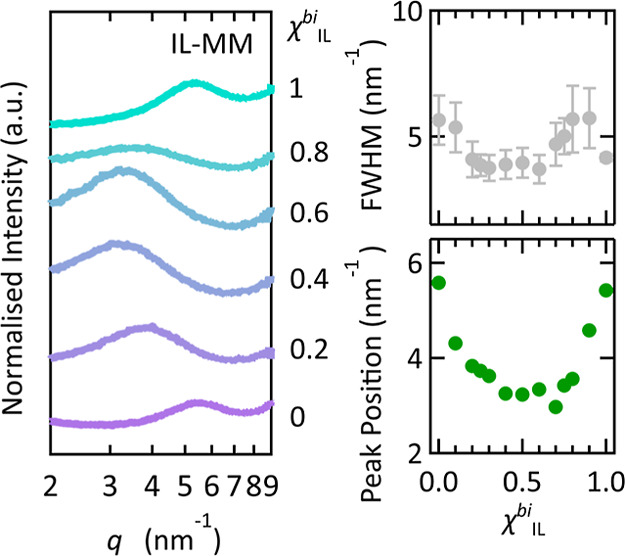
SAXS curves and fits of pseudobinary mixtures of the IL and MM. Only representative curves are shown; the full data set is in Supporting Information. The pseudobinary IL mole fraction (χ^bi^_IL_) is calculated by only considering the mole ratio between the IL and MM. That is, χ^bi^_IL_ = 0.5 means the mole ratio between IL/MM is 1:1, equivalent to a mole ratio among IL/DMB/NE = 1:1:1.

To confirm that our rationally designed molecular diluent has the desired effect on bulk viscosity while preserving the liquid nanostructure of the model IL, we have also performed viscosity measurements of representative IL–MM mixtures. Figure 5 clearly demonstrates the significant variation of viscosity of these mixtures, allowing continuous tuning over 3 orders of magnitude by simply varying the IL to MM ratio. We also applied a global fit of the experimental data by combining the Grunberg–Nissan mixing rule with the Vogel–Fulcher–Tammann temperature dependence (detailed in the Supporting Information).^23^ The global fitting parameter, which can be interpreted as viscosity at infinite temperature, was identical within uncertainty for the IL and MM, suggesting that their molecular similarities are also reflected in the temperature dependence of their bulk viscosity. The fitted Grunberg–Nissan interaction parameter, *G*, is negative and large, indicating significant negative deviations from ideal viscosity mixing. This reflects the disruption of intermolecular associations within each pure component, supporting the interpretation of IL–MM segregation.^24^

**Figure 5:**
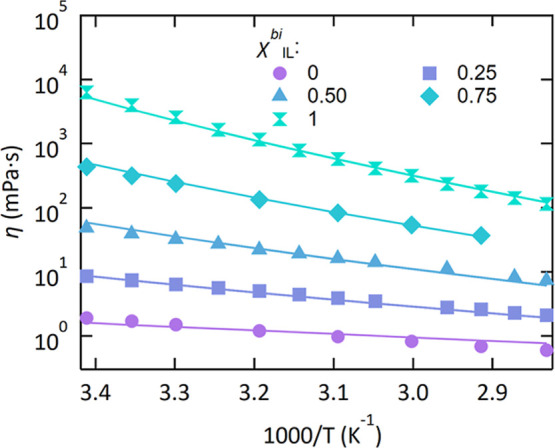
Experimental and fitted bulk viscosity of IL, MM, and their pseudobinary mixtures. Viscosity varies across 3 orders of magnitude, and a global fit was achieved using all data points.

We have also employed pulsed-field gradient NMR to probe the self-correlated diffusion coefficient of different species within these multicomponent mixtures (details in the Supporting Information). We observed a strong correlation between the average self-diffusion coefficient and the bulk viscosity. However, NE often diffused 5–10 times faster than other species in the mixture. The SAXS data show that NE occupies the same structural role as propionate because the liquid nanostructure is primarily dictated by molecular shape, size, and topology, which NE was designed to replicate. The dynamics, however, are governed not only by these topological factors but also by the interactions that each species experiences. Propionate, as a charged species, is coulombically bound within an ionic network. As a result, its motion is electrostatically coupled to that of the choline cations, giving rise to slower correlated self-diffusion. In contrast, NE carries no charge and is, therefore, not subject to these constraints. Similarly, we hypothesize that the slower diffusion of DMB reflects its participation in hydrogen-bonded networks, a constraint to which NE is also not subject. Therefore, NE can exchange local environments and escape transient cages far more readily, resulting in markedly faster diffusion, despite its structural equivalence to propionate. Structured liquids are known to exhibit diffusion-viscosity decoupling,^25,26^ and this clearly shows that distinct local environments are present in our IL–MM mixtures. This behavior underscores that the mimic replicates the topological contribution to structure while removing the Coulombic contribution to dynamics.

## Conclusion and Outlook

Ionic liquids are frequently limited in practical applications by their intrinsically high viscosities, which hinder mass and charge transport as well as their processability. The use of low-viscosity molecular diluents is a common strategy to address this challenge. However, such approaches often come at the cost of diminished functionality and the disruption of the characteristic nanostructure of ionic liquids. This lack of understanding of nanostructures in IL-based mixtures, especially with molecular diluents, greatly limits our ability to design new functional liquid mixtures. In this work, we have designed a model system by engineering a molecular diluent with structural similarity to the target ionic liquid (i.e., the molecular mimic). We further demonstrate that, by choosing an appropriate diluent such as the MM, it is possible to tune the viscosity of IL-based mixtures over 3 orders of magnitude while sustaining its characteristic nanostructure. While further studies are needed to gain atomic insights, our work clearly outlines a new strategy for designing functional IL–diluent systems. Beyond its practical implications, this model system also provides a platform to probe the role of Coulombic interactions in sustaining local order and governing bulk liquid properties, offering broader insights into the physics of complex fluids.

## Supplementary Material



## Notes

The Supporting Information is available free of charge at https://pubs.acs.org/doi/10.1021/acs.jpcb.6c03434. Full data sets supporting the findings of this study are available from the Oxford Research Archive under the same title.

Extended data sets; details of fitting procedures; complete pulsed-field gradient diffusion NMR data set; and ab initio calculations of molecular topology for the IL and MM (PDF)
