# Supplementary material for: Tailored Diluent for Nanostructure and Viscosity Control in an Ionic Liquid
Source: J Phys Chem B. 2026 Jul 27;130(31):8113–8. doi: 10.1021/acs.jpcb.6c03434 (PMC13455056; doi:10.1021/acs.jpcb.6c03434)
Supplement: Supplementary file 1 [file jp6c03434_si_001.pdf]

Supporting Information for

Tailored Diluent for Nanostructure and Viscosity Control in an Ionic Liquid

Shurui Miao*[1], Yilang Chen[1], Frederik Philippi*[2], Thomas Zinn[3], Kuno Schwärzer[4], Jia-Fei Poon[5], Lauren Matthews[6], Margarida Costa Gomes[2]

1. Physical and Theoretical Chemistry Laboratory, Department of Chemistry, University of Oxford, Oxford, OX1 2JD, UK

2. Laboratoire deChimie de l'ENS Lyon, CNRS and Université de Lyon, Lyon, 69007, France

3. Diamond Light Source, Diamond Light Source Ltd., Harwell Campus, Didcot OX11 0DE, UK

4. Jülich Centre for Neutron Science (JCNS-1), Forschungszentrum Jülich GmbH, Wilhelm-Johnen-Straße, 52428 Jülich, Germany

5. European Spallation Source, Box 176, SE-221 00, Lund, Sweden

6. ISIS Neutron and Muon Source, Rutherford Appleton Laboratory, Harwell Campus, Didcot, OX11 0QX, UK

Contents

Materials S2

Choline hydroxide-d_{13} S2

Nitroethane-d_5 ..... S4

3,3-dimethylbutan-1-ol-d_{14} S6

Small Angle X-ray Scattering (SAXS) S9

Temperature Dependence of SAXS Curves S10

SAXS Fitting S11

Small Angle Neutron Scattering (SANS) S12

Viscosity and Fitting S13

Diffusion NMR S15

Volume Calculation S17

References S19

Materials

Choline hydroxide-*d_{13}*

All chemicals were used as received unless otherwise stated. Deuterated ethylene oxide (D4, 98% stabilized with 0.1% hydroquinone) was purchased from Eurisotop and dried over CaH_2 before use. Trimethylammonium chloride (98%) and methanol (≥99.8%) were purchased from Sigma Aldrich. Amberlite IRA-402 (Cl^--form) was purchased from ThermoScientific and converted into the OH^--form as described below.

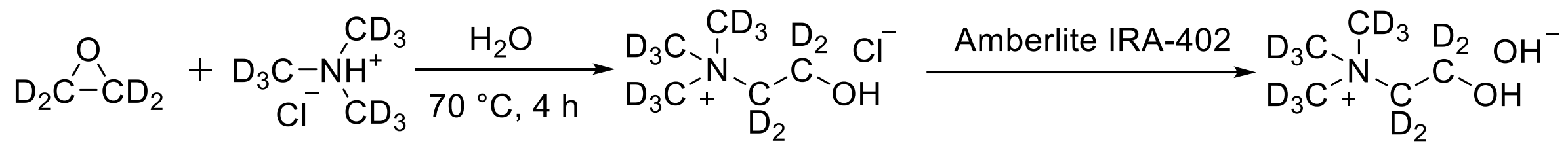

Synthesis of choline chloride-*d_{13}*: Tetrabutylammonium chloride-*d_9* (9.90 g, 94.6 mmol, 1.0 equiv.) was dissolved in 25 mL of water and degassed under vacuum at room temperature. The Schlenk flask was then sealed under vacuum and connected to a second sealed and evacuated Schlenk flask containing deuterated ethylene oxide (4.56 g, 94.7 mmol, 1.001 equiv.) via a Vigreux column and a short glass bridge under static vacuum. The flask containing the tetrabutylammonium chloride was heated to 70 °C and then the deuterated ethylene oxide was slowly transferred to it by opening the second flask. A lukewarm water bath was placed under the ethylene oxide flask to ensure complete evaporation. The reaction mixture was then stirred for 5 hours at 70 °C. Once the reaction was complete all volatile components were distilled off and the solid residue was dried under high vacuum and then recrystallized from isopropanol top yield 10.6 g (69.3 mmol, 73%) of choline chloride-*d_{13}* as colorless crystals. The deuteration degree was determined using ^{1}H-NMR spectroscopy with imidazole as an internal standard to be >99.5%.

Synthesis of choline hydroxide-d_{13}: A glass column with 5 cm a diameter of 5 cm was filled with Amberlite IRA-402 up to a height of 50 cm. The ion exchange resin was then washed with 5% aqueous NaOH solution until a test with $AgNO_3$ dissolved in diluted HNO_3 showed that no more chloride anions were eluted from the column. The resin was then washed with distilled water until pH = 7. The choline chloride-*d_{13}* (9.2 g, 60.5 mmol) from the last reaction step was dissolved in 100 mL of a 1:1 mixture of distilled water and methanol and slowly passed through the ion exchange column with the same solvent mixture. All fractions with a pH>8 were collected and combined to yield 317 g of choline hydroxide-*d_{13}* solution. Titration with 0.01 M HCl showed that the resulting solution had a concentration of 0.169 mmol/g (corresponds to 7.2 g, 53.5 mmol, 88% of choline hydroxide-*d_{13}*). Ion chromatography showed a residual chloride ion concentration of 0.0015 mmol/g.

Characterisation: NMR spectra were collected on a *Bruker* Avance NEO 600 MHz spectrometer with an *Inverse* 5 mm H/F-C/N-D CryoProbe. The samples were dissolved either in D_2O (^{1}H-NMR) or H_2O (^{2}H-NMR) and the measurements were conducted at 295 K. Ionchromatography (IC) was performed on a Metrohm IC 850 machine with a Metrosep A SUPP 7-250 column using 3.6 mM Na_2CO_3 as eluent. 1001 mg of the choline hydroxide-*d_{13}* solution were evaporated to dryness and dissolved in 5 mL of Milli-Q-water via ultrasonication for one hour followed by filtration through a 0.45 µm syringe filter.

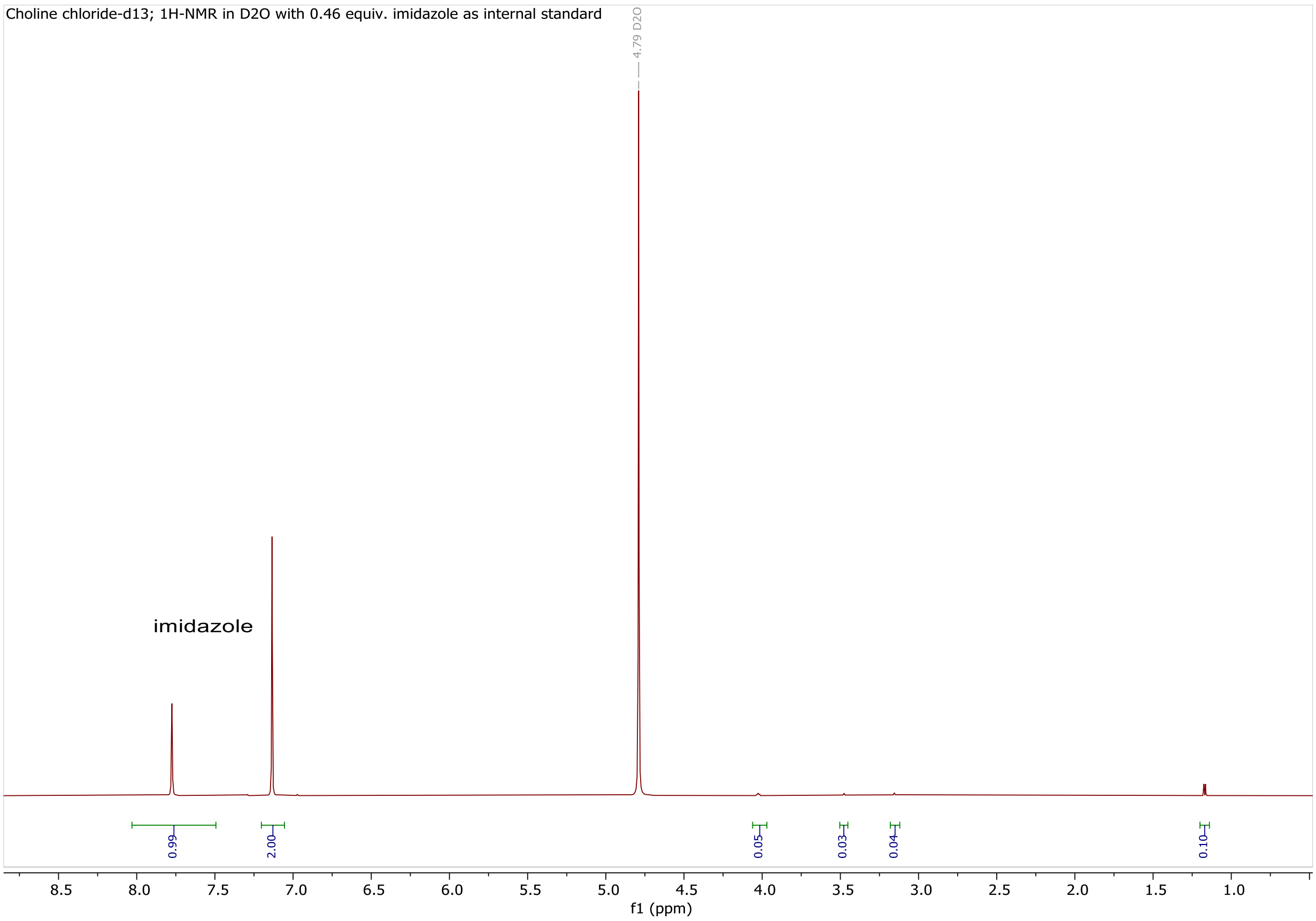

^{1}H-NMR of choline chloride-d_{13} in D_2O with imidazole as an internal standard.

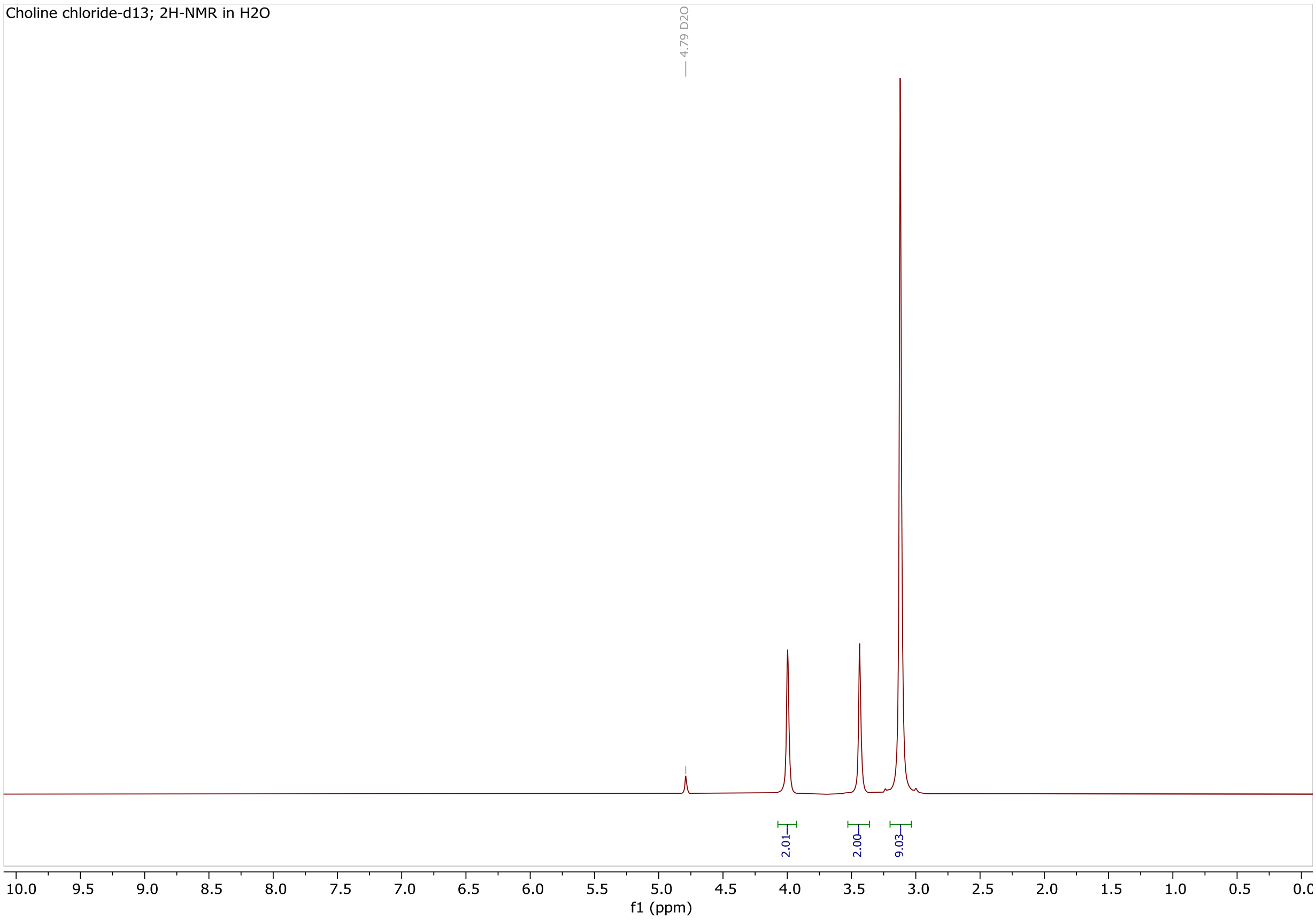

^{2}H-NMR of choline chloride-d_{13} in H_2O.

Nitroethane-*d*$_5$

All chemicals were used as received unless otherwise stated. Bromoethane-*d*$_5$ (99% D) was purchased from Armar Isotopes. Silver nitrite (99%) and diethyl ether (anhydrous, ≥99.0%) were purchased from Sigma-Aldrich.

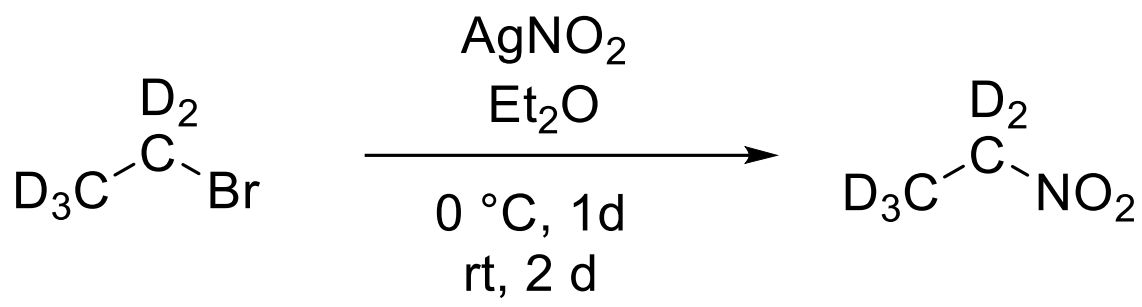

Nitroethane-*d*$_5$ was synthesized according to a slightly modified literature procedure:[1] Silver nitrite (26.3 g, 171 mmol, 1.5 equiv.) was suspended in 40 mL of dry diethyl ether inside an argon filled glovebox. The flask was sealed, removed from the glovebox, connected to a Schlenk line and cooled to 0 °C. Then deuterated ethyl bromide (13.0 g, 114 mmol, 1.0 equiv.) was added dropwise under argon counterpressure. The reaction flask was sealed with a Teflon stopper, protected from light and stirred at 0 °C for 1 day and then at room temperature for 2 more days. After the reaction was completed, all volatile compounds were distilled into a second flask. To remove the diethyl ether and the ethyl nitrite that was formed as a side product, the mixture was cooled to −50 °C and placed under high vacuum (approximately 10^{-2} mbar) until the weight of the residue remained constant. The resulting deuterated nitroethane (6.60 g, 82.3 mmol, 72%) was stored under argon. All contact with air or moisture was avoided to prevent a back exchange of the deuterium atoms in the alpha-position. NMR spectra using tetrabromoethane as an internal standard showed a deuteration degree of >98% and 0.4 wt% of diethyl ether as well as small traces of deuterated ethyl nitrite as the main impurities.

Characterization: NMR spectra were collected on a *Bruker* Avance NEO 600 MHz spectrometer with an *Inverse* 5 mm H/F-C/N-D CryoProbe. The samples were dissolved either in $CDCl_3$ (^{1}H-NMR) or CH_2Cl_2 (^{2}H-NMR) and the measurements were conducted at 295 K.

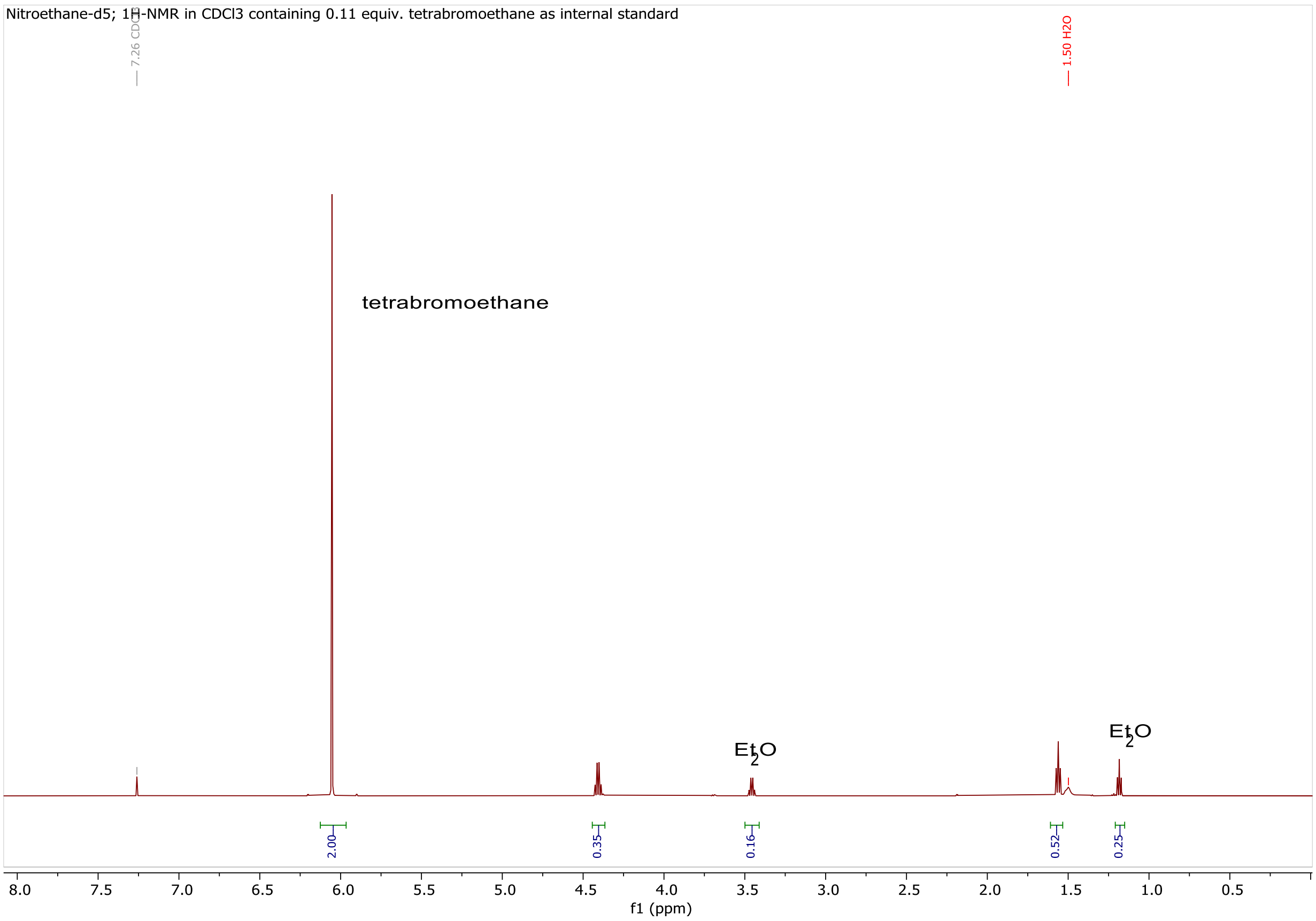

^{1}H-NMR of nitroethane-d_5 in $CDCl_3$ with tetrabromoethane as internal standard

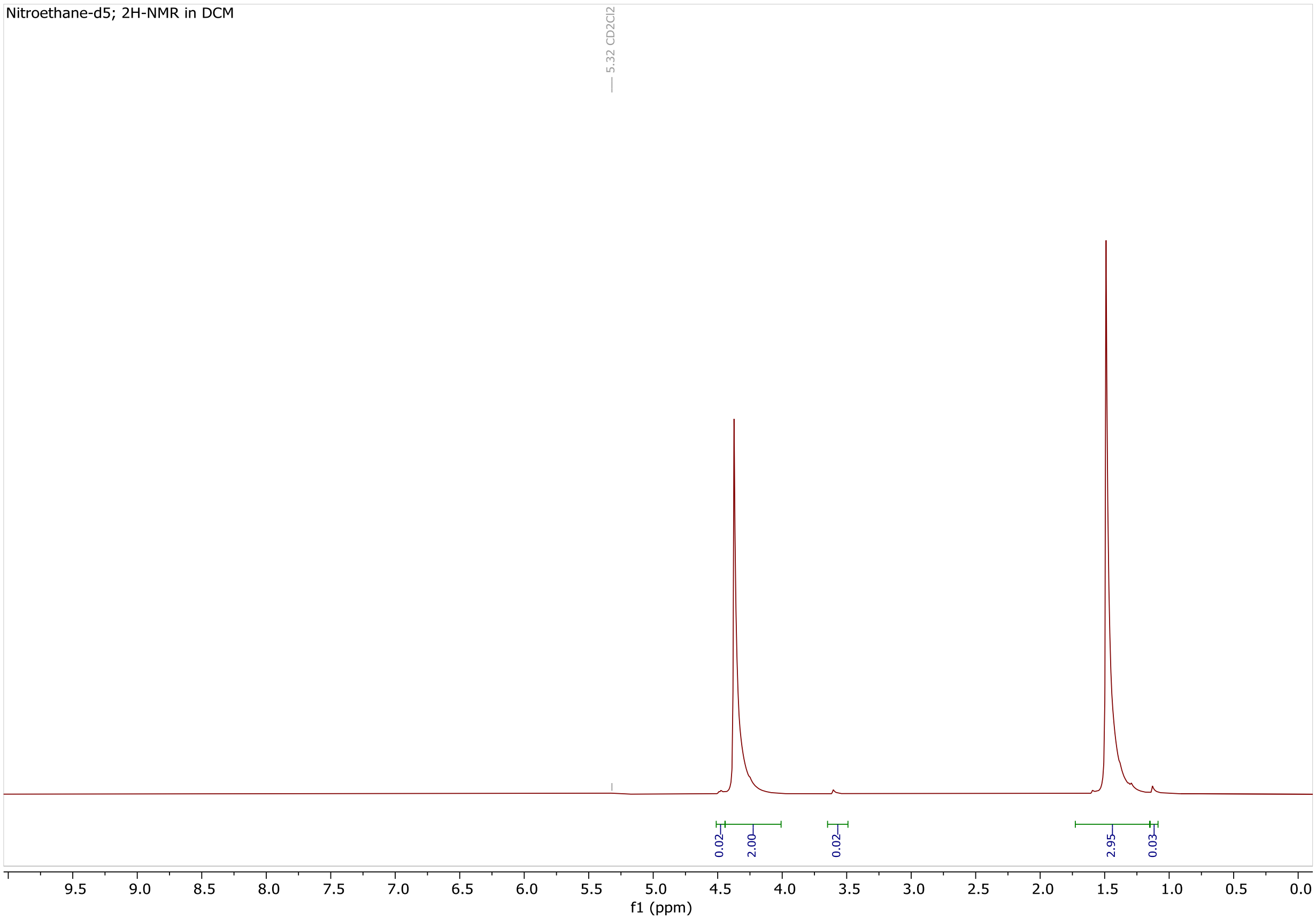

^{2}H-NMR of nitroethane-d_5 in CH_2Cl_2

3,3-dimethylbutan-1-ol-*d*$_{14}$

General. Reagents were purchased from commercial suppliers and used without further purification. Methyl 3,3-dimethylbutanoate was synthesized according to literature procedure.[2] NMR spectra were recorded on a Bruker AVANCE spectrometer (500 and 126 MHz for ^{1}H and ^{13}C NMR, respectively) or Varian Unity Inova (400 MHz for ^{1}H).

3,3-dimethylbutanoic acid-d$_{11}$. 3,3-dimethylbutanoic acid (20 g, 172 mmol), NaOD (17.7 g, 40 wt% in D_2O, 172 mmol), Pt/C (5 wt%, 1 g) and D_2O (200 mL) were loaded to a Parr reactor. After degassing with N_2 for 20 min, the vessel was sealed and allowed to stir at 220 °C for 3 days. The reaction mixture was acidified with 1M HCl, filtered and extracted with EtOAc (200 mL x 3). The combined organic phase was washed with brine (100 mL), dried over $MgSO_4$, filtered and evaporated under reduced pressure to afford the title compound as colourless oil (20.9 g, 96%). The aforementioned reaction was repeated for another two cycles to afford the title compound in >98%D. ^{1}H NMR (400 MHz, $CDCl_3$) δ 2.09 (s, 0.02H, residual), 1.00 (s, 0.09H, residual).^{13}C NMR (126 MHz, $CDCl_3$) δ 179.2, 47.0 (m), 29.8, 28.5 (m).

3,3-dimethyl -1-butanol-d$_{14}$. To a solution of methyl 3,3-dimethylbutanoate-d$_{11}$ (10 g, 79 mmol) in anhydrous diethyl ether (90 mL) was added $LiAlD_4$ (5 g, 118 mmol) at 0 °C under N_2. After stirring for 19h, the reaction was quenched with 3M DCl in D_2O and extracted with diethyl ether (100 mL x 3). The combined organic phase was dried over $MgSO_4$, filtered and evaporated under reduced pressure. The residue was purified by distillation under reduced pressure to afford the title compound as colourless liquid (7.4 g, 81%, >98%D). ^{1}H NMR (500 MHz, $CDCl_3$) δ 1.25 – 1.19 (complex, 0.02H, residual), 0.86 (m, 0.09H, residual). ^{13}C NMR (126 MHz, $CDCl_3$) δ 59.2 (m), 45.2 (m), 29.2 – 28.1 (complex, 2C).

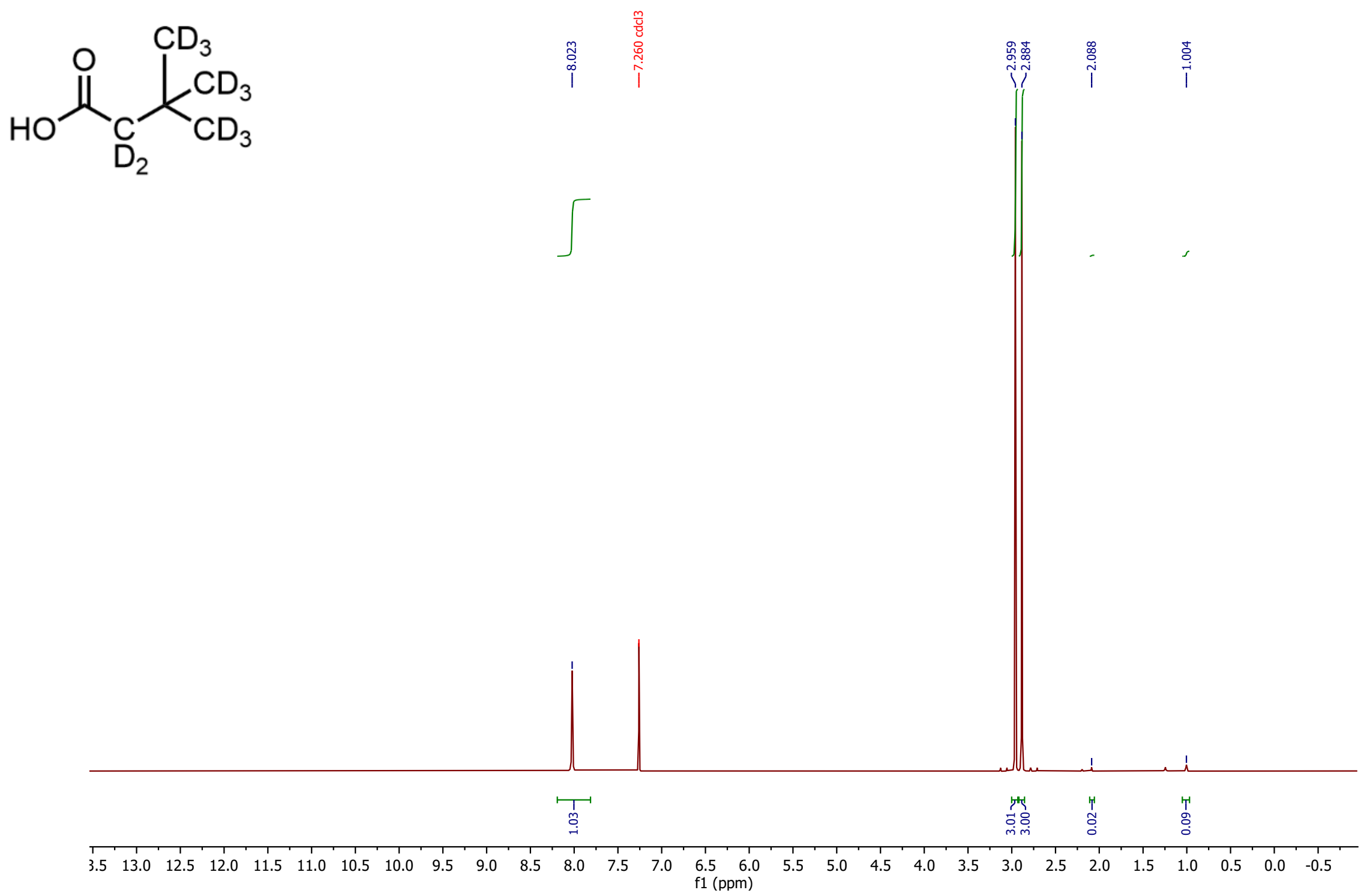

^{1}H NMR spectrum of 3,3-dimethylbutanoic acid-d$_{11}$ in the presence of equimolar DMF.

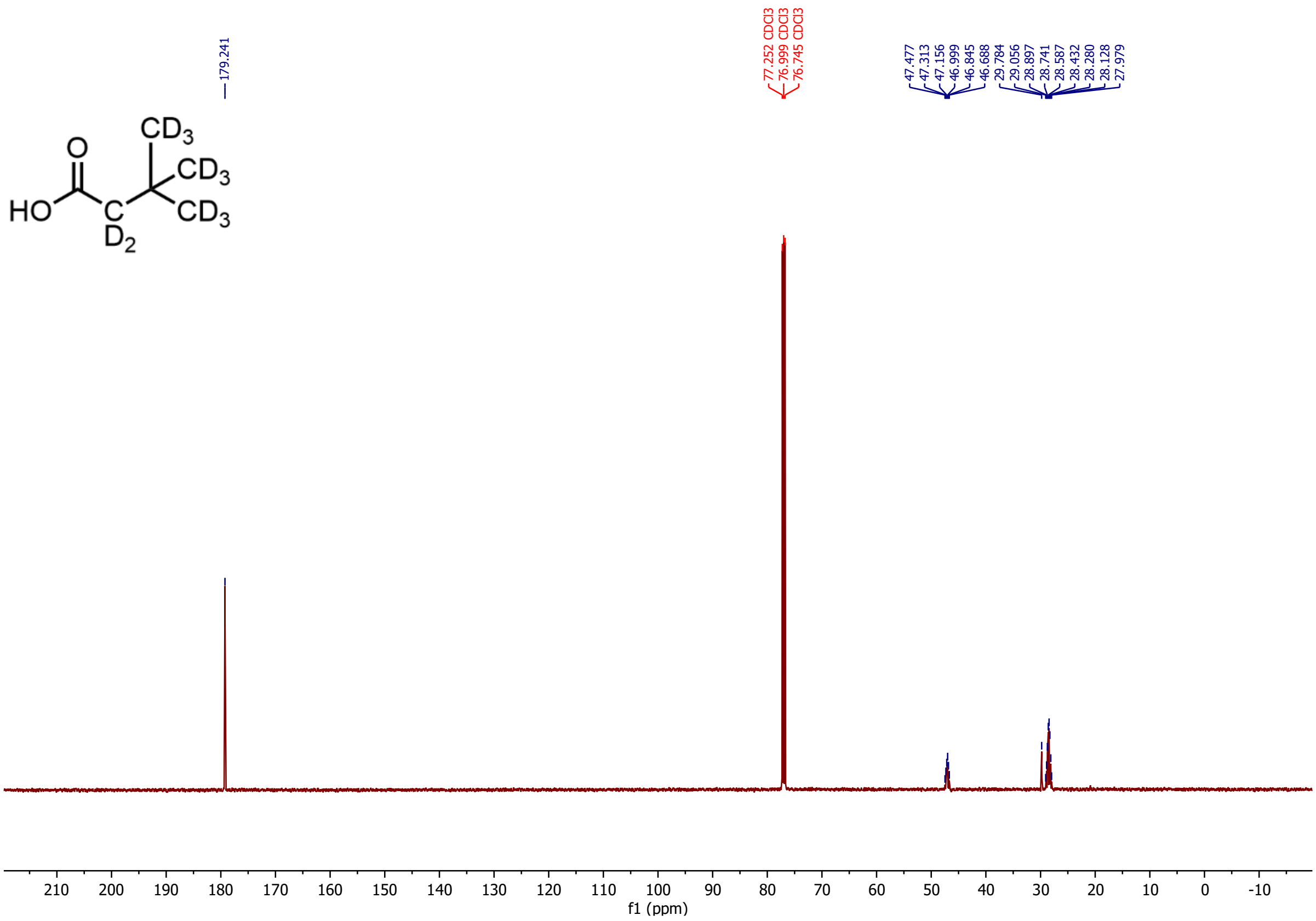

^{13}C NMR spectrum of 3,3-dimethylbutanoic acid-d_{11}.

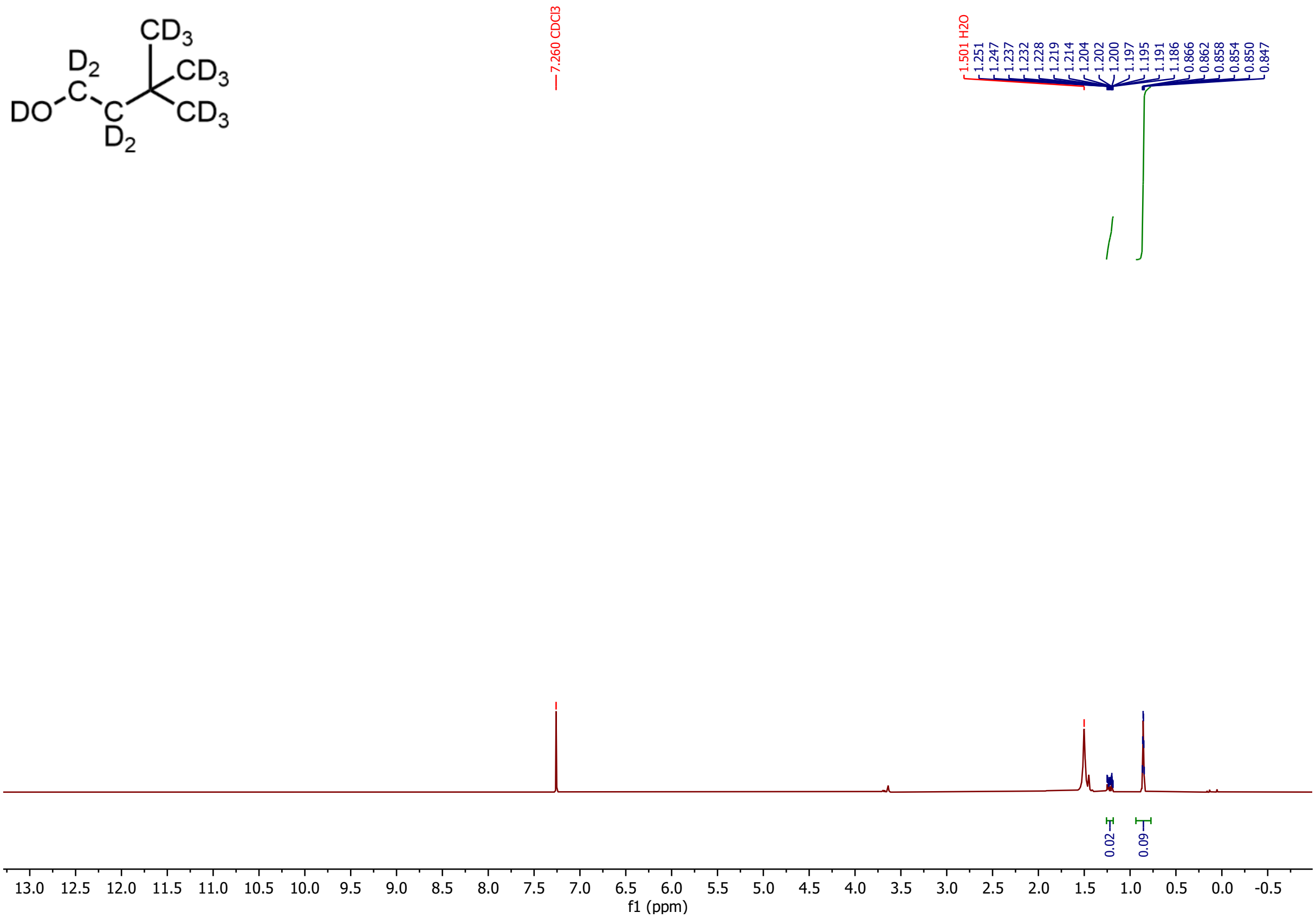

^{1}H NMR spectrum of 3,3-dimethyl -1-butanol-d_{14}.

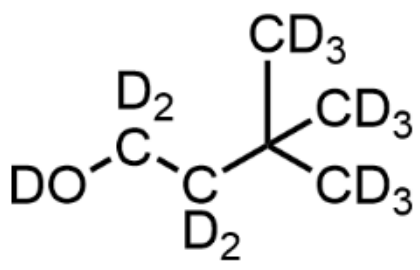

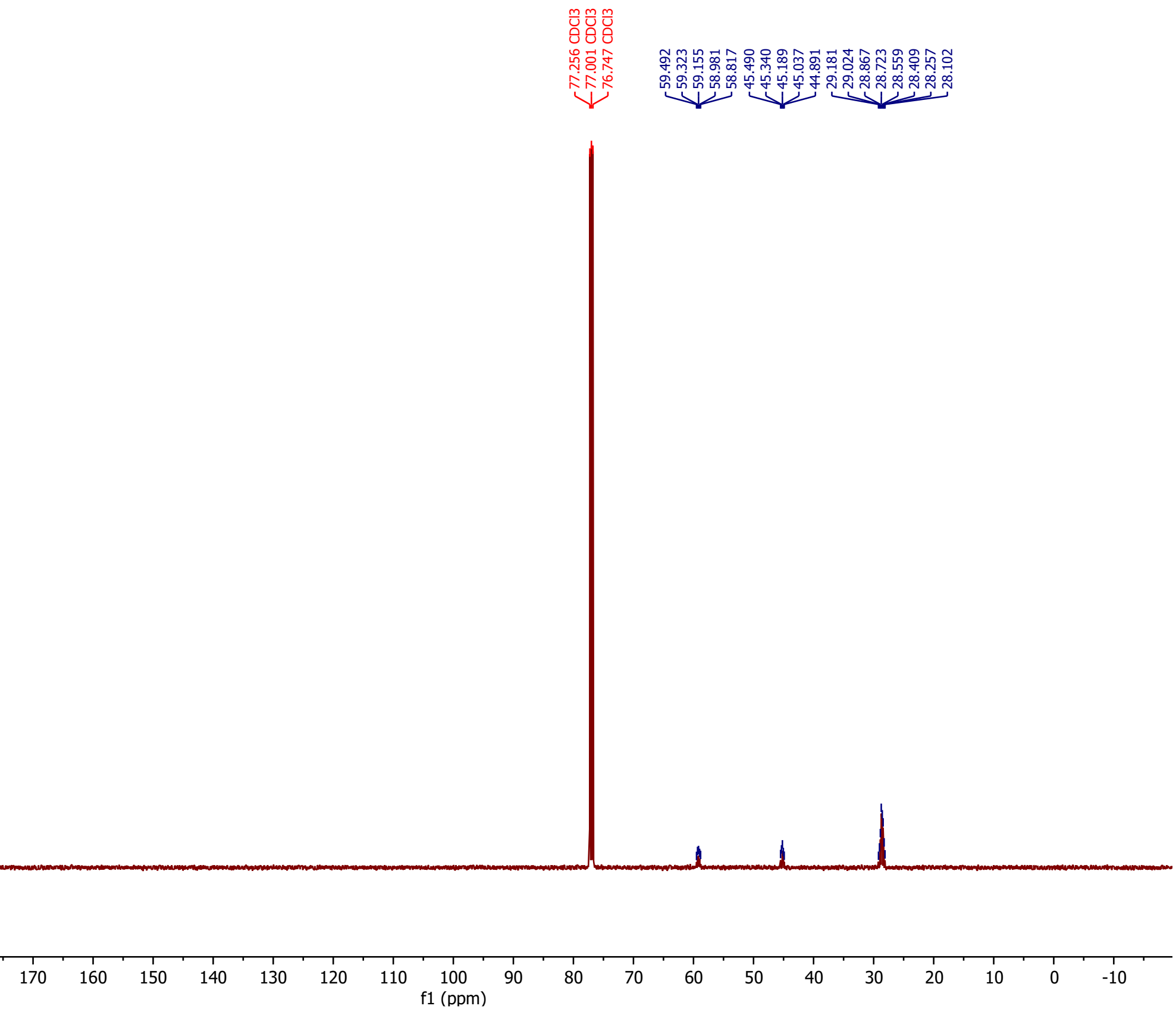

^{13}C NMR spectrum of 3,3-dimethyl -1-butanol-d$_{14}$.

Small Angle X-ray Scattering (SAXS)

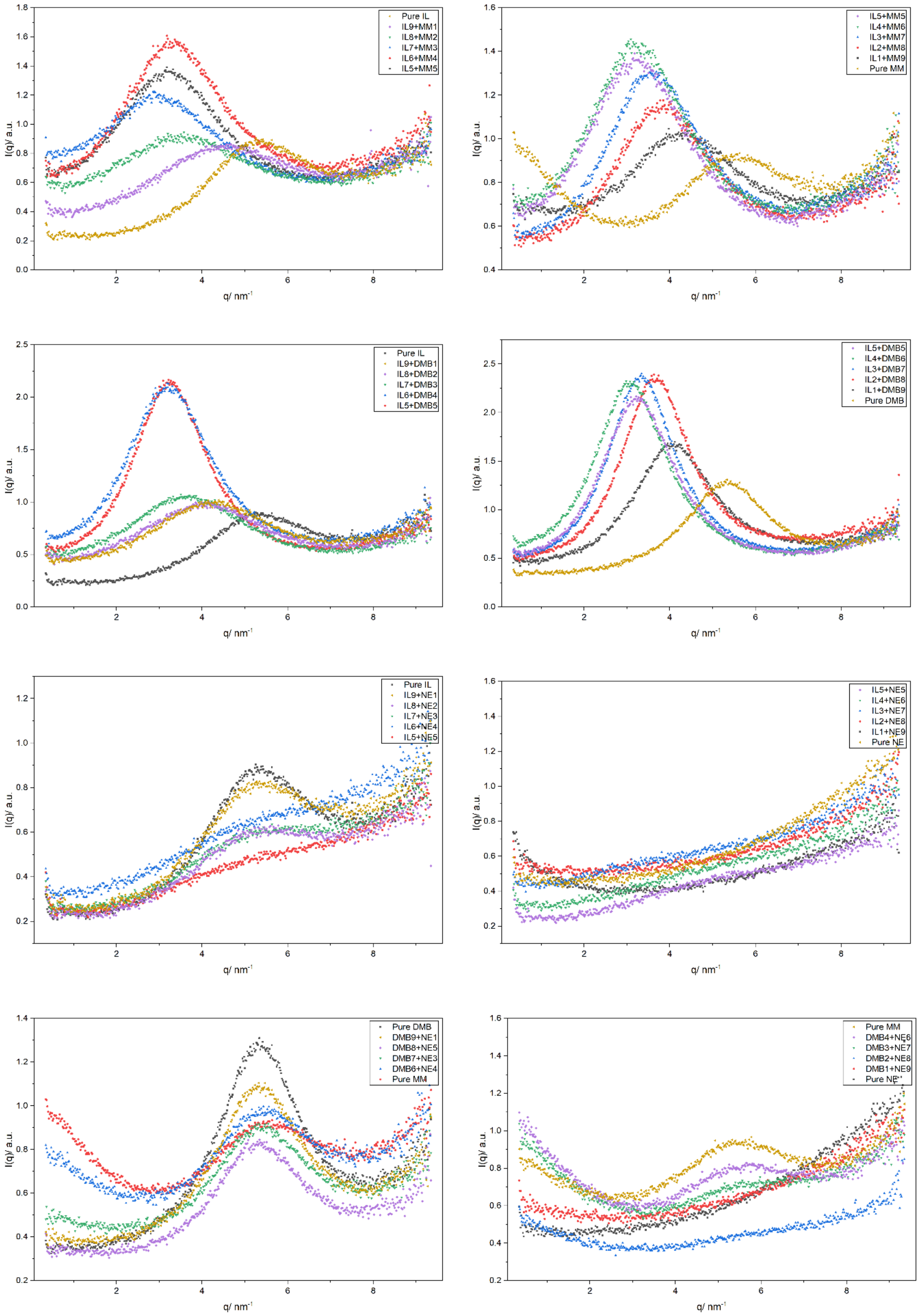

Figure S1. Experimental SAXS curves of all liquids studied at 30 °C

Temperature Dependence of SAXS Curves

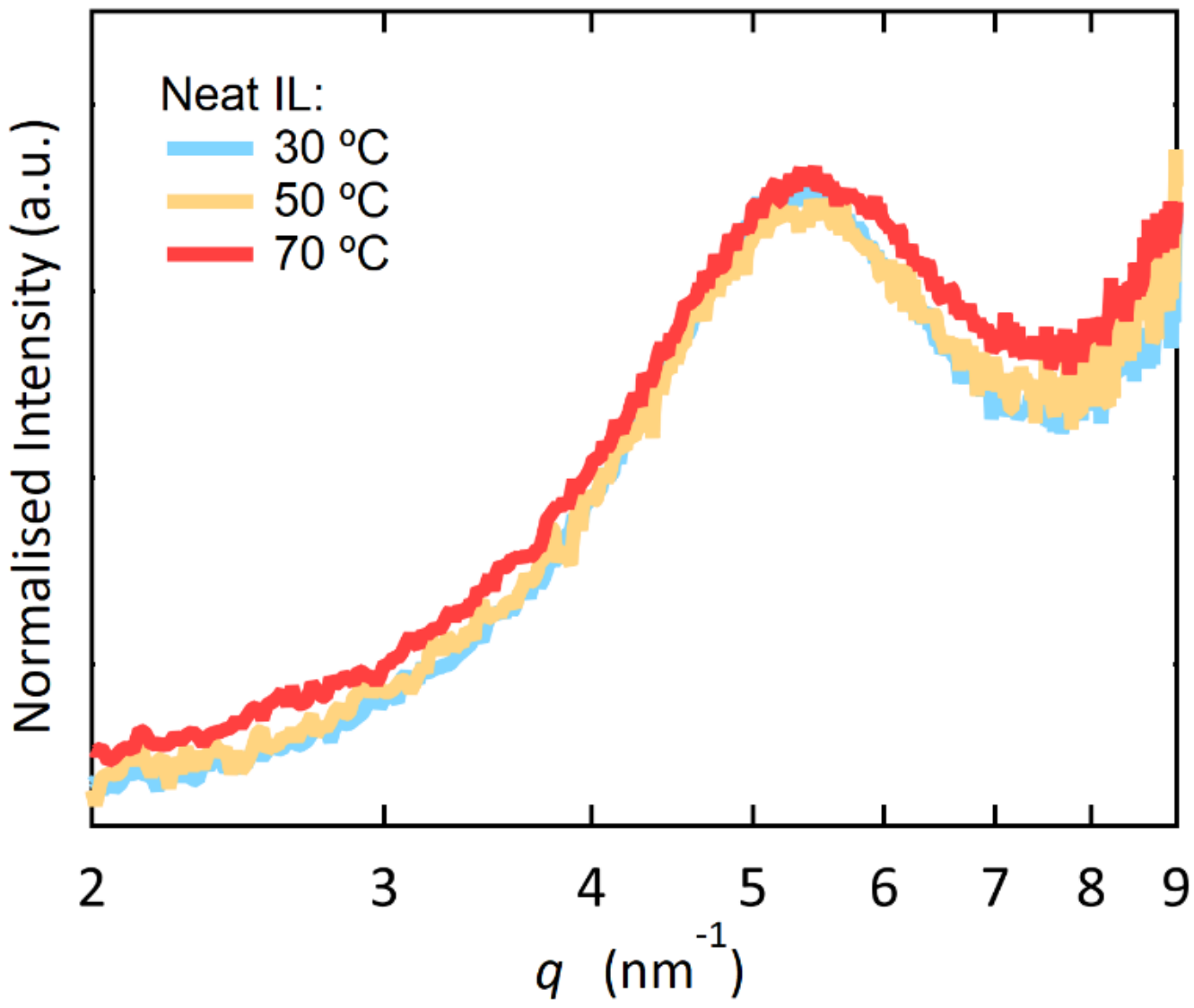

Figure S2. Experimental SAXS curves of the neat IL measured at 3 temperatures.

A characteristic feature of the prepeak is that it grows in intensity with temperature, exhibiting the anti-Debye-Waller behaviours. Therefore, we have acquired SAXS curves of all compositions at 30, 50, and 70 °C. Although the temperature dependence is weak, the observed scattering peak in the neat IL shows no noticeable broadening with increasing temperature. This confirms that the model IL possess a prepeak. Therefore, scattering peaks throughout this study are either referred to as the prepeak or prepeak-like features. Due to this weak temperature dependence, all discussion presented are based on scattering curves obtained at 30 °C (minimal risk of compositional change due to evaporation). The complete dataset can be accessed via the Oxford University Research Archive.

SAXS Fitting

The Teubner-Strey model (TS model) is commonly applied to similar systems to quantify correlation length and periodicity. However, we cannot perform consistent and unambiguous fit to all our SAXS curves using this model. This is due to the lack of an experimental baseline, the *q*-independent scattering of our samples, which is strongly correlated with other parameters of the TS model. Therefore, we have adopted a more general model to describe the observed scattering peak based on the Lorentzian function:

$$I(q) = \frac{A}{\left(q - q_{peak}\right)^2 + B}$$

where *A* governs the fitted intensity, q_{peak} is the fitted peak position, and *B* controls the width of the fitted peak. The effective full width at half maximum (FWHM) can be obtained as $2\sqrt{B}$. The Lorentzian function is also sensitive to the baseline. However, we can reduce this dependence by limiting the fitted range to a narrower window such as q_{peak} ± 1 nm^{-1} (i.e., a 2 nm^{-1} window). This necessarily introduces larger uncertainties to the fitted values but effectively eliminates the baseline dependence. Therefore, q_{peak} and the FWHM can be interpreted as a set of consistent and model-free features of the scattering peak. This allows quantitative comparison and provides insights regarding the evolution of self-assembled nanostructures.

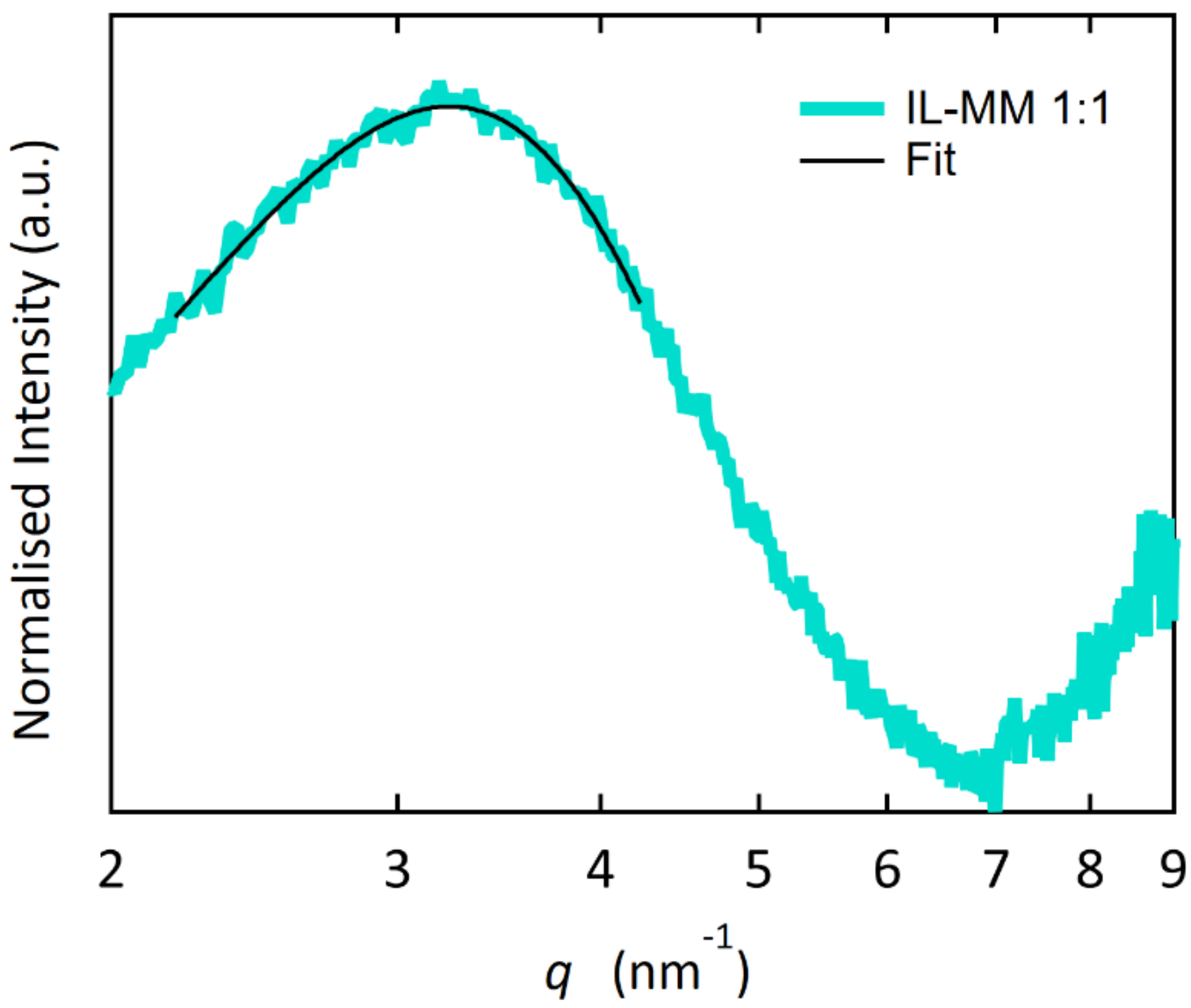

Figure S3. Experimental SAXS curve of the IL and its Lorentzian fit over the q_{peak} ± 1 nm^{-1} region. While the fit is not perfect, it allows consistent treatment of our entire datasets and enable quantitative comparisons.

Small Angle Neutron Scattering (SANS)

The SAXS pattern of the molecular mimic (equimolar mixture of nitroethane and 3,3-dimethyl-1-butanol) showed a rise in scattering intensity at the low-*q* limit SAXS (Figure S1). Similarly, many mixtures of IL and molecular species resulted in shift in the prepeak-like feature toward the lower-*q* region. Therefore, to confirm that no large aggregates or critical behaviours are present in our mixtures, we performed SANS with deuterated chemicals, results are shown in Figure S4.

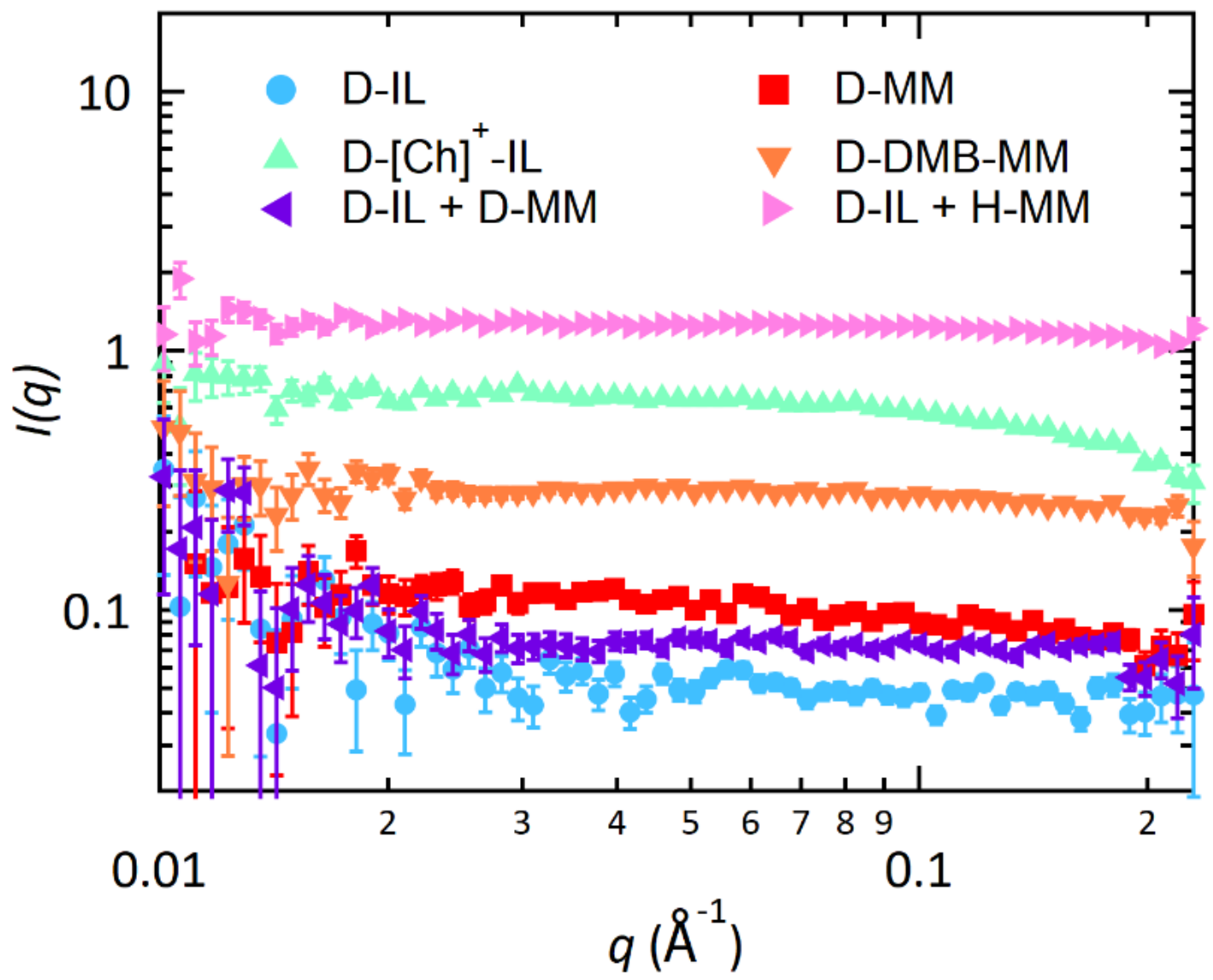

Figure S4. Experimental SANS curves of neat IL and MM (equimolar mixture of nitroethane and 3,3-dimethyl-1-butanol), as well as the 1:1 mixture of IL and MM. The 6 contrasts are (left to right, top to bottom): i) fully deuterated neat IL; ii) fully deuterated MM; iii) cation deuterated neat IL; iv) 3,3-dimethyl-1-butanol deuterated MM; v) fully deuterated 1:1 mixture of IL and MM; vi) fully deuterated IL + hydrogenous MM (1:1).

All SANS curves have plateaued to a constant scattering intensity before reaching the low-*q* limit. This confirms that no critical fluctuation is present in the bulk. The SANS curves of cation deuterated neat IL (▲) and the fully deuterated MM (■) showed small increase in scattering intensity around 0.2 $Å^{-1}$ towards lower-*q*, consistent with SAXS, suggesting that some small self-assembled structures are present. This can be attributed to the known presence of alkanol clustering in the MM, and the charged networks in IL.

Viscosity and Fitting

Table S1. Experimental viscosity η_{exp} and density ρ_{exp} as a function of temperature T, mole fraction ionic liquid χ_{IL}, and mole fraction molecular mimic χ_{MM}.

T / K	x_{IL}	x_{MM}	η_{exp} / mPa s	ρ_{exp} / g cm^{-3}	T / K	x_{IL}	x_{MM}	η_{exp} / mPa s	ρ_{exp} / g cm^{-3}
293.15	1	0	6349	1.0700	333.15	0	1	0.82	0.8597
298.15	1	0	3956	1.0671	343.15	0	1	0.69	0.8500
303.15	1	0	2584	1.0643	353.15	0	1	0.60	0.8402
313.15	1	0	1152	1.0588	293.15	0.25	0.75	8.5	0.9387
323.15	1	0	576	1.0536	298.15	0.25	0.75	7.4	0.9351
333.15	1	0	313	1.0482	303.15	0.25	0.75	6.4	0.9312
343.15	1	0	179	1.0429	313.15	0.25	0.75	5.0	0.9231
353.15	1	0	114	1.0377	323.15	0.25	0.75	3.9	0.9145
348.15	1	0	142	1.0404	343.15	0.25	0.75	2.6	0.9006
338.15	1	0	234	1.0457	353.15	0.25	0.75	2.1	0.8940
328.15	1	0	412	1.0511	348.15	0.25	0.75	2.3	0.8981
318.15	1	0	786	1.0565	338.15	0.25	0.75	2.8	0.9061
308.15	1	0	1641	1.0618	328.15	0.25	0.75	3.5	0.9141
298.15	1	0	3836	1.0673	318.15	0.25	0.75	4.4	0.9220
293.15	1	0	6122	1.0701	308.15	0.25	0.75	5.6	0.9299
303.15	1	0	2527	1.0645	298.15	0.25	0.75	7.3	0.9379
293.15	0.75	0.25	436	1.0270	293.15	0.25	0.75	8.5	0.9418
298.15	0.75	0.25	315	1.0239	293.15	0	1	1.9	0.8973
303.15	0.75	0.25	236	1.0208	298.15	0	1	1.7	0.8927
313.15	0.75	0.25	134	1.0146	303.15	0	1	1.5	0.8880
323.15	0.75	0.25	83	1.0085	313.15	0	1	1.2	0.8787
333.15	0.75	0.25	54	1.0022	323.15	0	1	0.98	0.8693
343.15	0.75	0.25	37	0.9957					
293.15	0.5	0.5	48	0.9843					
298.15	0.5	0.5	39	0.9808					
303.15	0.5	0.5	32	0.9772					
313.15	0.5	0.5	22	0.9701					
323.15	0.5	0.5	16	0.9626					
353.15	0.5	0.5	7.3	0.9281					
348.15	0.5	0.5	8.2	0.9323					
338.15	0.5	0.5	11	0.9409					
328.15	0.5	0.5	14	0.9502					
318.15	0.5	0.5	19	0.9584					
308.15	0.5	0.5	27	0.9665					

The viscosity varies over several orders of magnitude. A global fit of the experimental data was performed using the equation below, which combines the Grunberg-Nissan mixing rule with Vogel-Fulcher-Tammann temperature dependence. Notably, the parameter for the viscosity at infinite temperature η_0 was not significantly different for the molecular mimic and the ionic liquid.

$$\ln\eta = \ln\eta_0 + \frac{x_{IL}B_{IL}}{T - T_{0,IL}} + \frac{x_{MM}B_{MM}}{T - T_{0,MM}} + x_{IL}x_{MM}G_{IL-MM}$$

The numerical values for the fit parameters are summarised in Table S2. With these parameters and their numerical precision as reported in the table, the average (median) absolute deviation from the experimental data is 8% (7%).

Table S2. Summary of global fit parameters for the viscosity data.

	IL	MM
η_0	0.038 mPa s	
B	1519 K	911 K
T_0	165 K	50 K
G_{IL-MM}	-1.83	

Diffusion NMR

Table S3. Diffusion coefficients from PFGSTE diffusometry. A Bruker Avance III 400 MHz spectrometer equipped with 5 mm CryoProbe Prodigy probe was used for low viscosity samples, indicated blue. Samples with high viscosity were measured on a Bruker Avance III 400 MHz spectrometer equipped with BBIDIff probe, indicated red. The reported values are averages over all discernible peaks in the spectra and measured across the two probe heads for green entries. The alcohol moiety is listed as a separate entity since due to exchange it is not possible to discern butanol/choline/water traces.

T / K	x_{IL}	x_{MM}	Species	D / cm^2 s^{-1}
303.15	1	0	OH	7.4E-09
303.15	1	0	choline	7.1E-09
303.15	1	0	propionate	7.2E-09
323.15	1	0	OH	2.9E-08
323.15	1	0	choline	2.7E-08
323.15	1	0	propionate	2.9E-08
343.15	1	0	OH	9.7E-08
343.15	1	0	choline	9.3E-08
343.15	1	0	propionate	9.5E-08
303.15	0.75	0.25	choline	4.7E-08
303.15	0.75	0.25	DMB	7.5E-08
303.15	0.75	0.25	propionate	5.3E-08
323.15	0.75	0.25	OH	2.8E-07
323.15	0.75	0.25	choline	1.4E-07
323.15	0.75	0.25	DMB	2.2E-07
323.15	0.75	0.25	propionate	1.5E-07
343.15	0.75	0.25	OH	6.5E-07
343.15	0.75	0.25	choline	3.1E-07
343.15	0.75	0.25	DMB	5.0E-07
343.15	0.75	0.25	propionate	3.3E-07
303.15	0.5	0.5	OH	4.5E-07
303.15	0.5	0.5	NE	1.9E-06
303.15	0.5	0.5	choline	2.8E-07
303.15	0.5	0.5	DMB	5.8E-07
303.15	0.5	0.5	propionate	3.0E-07
323.15	0.5	0.5	OH	9.2E-07
323.15	0.5	0.5	choline	4.9E-07
323.15	0.5	0.5	propionate	5.3E-07
323.15	0.5	0.5	DMB	1.0E-06
343.15	0.5	0.5	OH	1.7E-06
343.15	0.5	0.5	DMB-OH	4.5E-06
343.15	0.5	0.5	choline	9.5E-07

343.15	0.5	0.5	propionate	1.0E-06
343.15	0.5	0.5	DMB	1.9E-06
303.15	0.25	0.75	OH	1.9E-06
303.15	0.25	0.75	NE	5.8E-06
303.15	0.25	0.75	choline	1.0E-06
303.15	0.25	0.75	DMB	1.9E-06
303.15	0.25	0.75	propionate	1.0E-06
323.15	0.25	0.75	OH	3.3E-06
323.15	0.25	0.75	NE	9.5E-06
323.15	0.25	0.75	choline	1.8E-06
323.15	0.25	0.75	propionate	1.9E-06
323.15	0.25	0.75	DMB	3.7E-06
343.15	0.25	0.75	OH	5.3E-06
343.15	0.25	0.75	NE	1.4E-05
343.15	0.25	0.75	choline	3.0E-06
343.15	0.25	0.75	propionate	3.1E-06
343.15	0.25	0.75	DMB	6.0E-06
303.15	0	1	NE	1.5E-05
303.15	0	1	OH	6.8E-06
303.15	0	1	DMB	6.8E-06
303.15	0	1	DMB/NE	1.0E-05
323.15	0	1	NE	2.1E-05
323.15	0	1	OH	1.1E-05
323.15	0	1	DMB	1.1E-05
343.15	0	1	NE	3.0E-05
343.15	0	1	DMB	1.7E-05
343.15	0	1	OH	1.8E-05

Volume Calculation

Ab initio calculations were performed using the ORCA software package, version 6.1.1.[3, 4] Initial geometries were fully optimised at the ωB97X-GD3(BJ)/def2-TZVPD level of theory,[5-11] employing the RIJCOSX approximation,[12, 13] together with the def2/J auxiliary basis set.[14] Tight self-consistent field convergence (TightSCF) and tight geometry optimisation criteria (TightOpt) were applied throughout. Analytical frequencies were calculated to verify stationary points as true minima.[15] Optimisations were performed in gas phase without symmetry constraints.

Wavefunction analysis was carried out using the Multiwfn software package, version 3.8, to obtain the molecular volumes enclosed by the 0.001 isosurface.[16-18] To this end, a grid spacing of 0.1 Bohr was used. The results are presented in Table S4.

Table S4. Volumes for the four constituents, calculated in isolation in the gas phase.

		Volume / $Å^3$
Molecular Mimic	Dimethylbutanol (DMB)	167.0
∑ = 262.9 $Å^3$	Nitroethane (NE)	95.9
Ionic Liquid	Choline	154.5
∑ = 262.6 $Å^3$	Propionate	108.1

The differences between ionic liquid and molecular mimic are small; both have a molecular volume around 158 mL/mol if additive behaviour is assumed. The additional negative charge on the anion slightly increases the volume enclosed by the 0.001 isosurface compared to nitroethane as expected, and *vice versa* for choline compared to dimethyl butanol. In the bulk liquid, coulombic compaction occurs, as discussed previously.[19] We performed additional calculations on choline propionate ion pairs as well as a molecular pair of dimethyl butanol and nitroethane. We used the GOAT algorithm implemented in ORCA to generate an ensemble of different orientations and conformations.[20-22] The 10 lowest energy conformers were then optimised at the ωB97X-GD3(BJ)/def2-TZVPD level of theory as described above. In addition, we performed the optimisations in the C-PCM implicit water model to gauge the dependence on the environment.[23] The results are summarised in Table S5. The difference in molecular volume between ionic liquid and molecular mimic is small, around 3.6% in gas phase and 2.7% in implicit solvation environment. In either case, the trend is consistent with the expected coulombic compaction, *i.e.* the higher cohesive energy of ionic systems.

Table S5. Volumes calculated for a single ion pair of the ionic liquid as well as the respective pair of molecules for the molecular mimic; comparison between gas phase and implicit solvation environment. Presented values are averages over the 10 lowest-energy conformers, the standard deviation is given in brackets.

	Volume / Å^3	
	Gas phase	C-PCM(Water)
Molecular Mimic (dimethyl butanol + nitroethane)	258.5(7)	260.6(5)
Ionic Liquid (Choline + propionate)	249.4(3)	253.7(6)

References

(1) Kornblum, N.; Ungnade, H. E. *Organic Syntheses*; 1963.
(2) ter Wiel, M. K. J.; Kwit, M. G.; Meetsma, A.; Feringa, B. L. Synthesis, stereochemistry, and photochemical and thermal behaviour of bis-tert-butyl substituted overcrowded alkenes. *Organic & Biomolecular Chemistry* **2007**, *5* (1), 87-96, 10.1039/B611070C. DOI: 10.1039/B611070C.
(3) Neese, F. The ORCA program system. *WIREs Computational Molecular Science* **2012**, *2* (1), 73-78. DOI: 10.1002/wcms.81.
(4) Neese, F. Software Update: The ORCA Program System—Version 6.0. *WIREs Computational Molecular Science* **2025**, *15* (2), e70019. DOI: 10.1002/wcms.70019.
(5) Rappoport, D. Property-optimized Gaussian basis sets for lanthanides. *The Journal of Chemical Physics* **2021**, *155* (12), 124102. DOI: 10.1063/5.0065611.
(6) Rappoport, D.; Furche, F. Property-optimized Gaussian basis sets for molecular response calculations. *The Journal of Chemical Physics* **2010**, *133* (13), 134105. DOI: 10.1063/1.3484283.
(7) Grimme, S.; Ehrlich, S.; Goerigk, L. Effect of the damping function in dispersion corrected density functional theory. *Journal of Computational Chemistry* **2011**, *32* (7), 1456-1465. DOI: 10.1002/jcc.21759.
(8) Grimme, S.; Antony, J.; Ehrlich, S.; Krieg, H. A consistent and accurate ab initio parametrization of density functional dispersion correction (DFT-D) for the 94 elements H-Pu. *The Journal of Chemical Physics* **2010**, *132* (15), 154104. DOI: 10.1063/1.3382344.
(9) Weigend, F.; Ahlrichs, R. Balanced basis sets of split valence, triple zeta valence and quadruple zeta valence quality for H to Rn: Design and assessment of accuracy. *Physical Chemistry Chemical Physics* **2005**, *7* (18), 3297-3305. DOI: 10.1039/b508541a.
(10) Najibi, A.; Goerigk, L. The Nonlocal Kernel in van der Waals Density Functionals as an Additive Correction: An Extensive Analysis with Special Emphasis on the B97M-V and ωB97M-V Approaches. *Journal of Chemical Theory and Computation* **2018**, *14* (11), 5725-5738. DOI: 10.1021/acs.jctc.8b00842.
(11) Chai, J.-D.; Head-Gordon, M. Systematic optimization of long-range corrected hybrid density functionals. *The Journal of Chemical Physics* **2008**, *128* (8), 084106. DOI: 10.1063/1.2834918.
(12) Neese, F. An improvement of the resolution of the identity approximation for the formation of the Coulomb matrix. *Journal of Computational Chemistry* **2003**, *24* (14), 1740-1747. DOI: 10.1002/jcc.10318.
(13) Neese, F.; Wennmohs, F.; Hansen, A.; Becker, U. Efficient, approximate and parallel Hartree–Fock and hybrid DFT calculations. A 'chain-of-spheres' algorithm for the Hartree–Fock exchange. *Chemical Physics* **2009**, *356* (1), 98-109. DOI: 10.1016/j.chemphys.2008.10.036.
(14) Weigend, F. Accurate Coulomb-fitting basis sets for H to Rn. *Physical Chemistry Chemical Physics* **2006**, *8* (9), 1057-1065. DOI: 10.1039/b515623h.
(15) Bykov, D.; Petrenko, T.; Izsák, R.; Kossmann, S.; Becker, U.; Valeev, E.; Neese, F. Efficient implementation of the analytic second derivatives of Hartree–Fock and hybrid DFT energies: a detailed analysis of different approximations. *Molecular Physics* **2015**, *113* (13-14), 1961-1977. DOI: 10.1080/00268976.2015.1025114.
(16) Lu, T.; Chen, F. Multiwfn: A multifunctional wavefunction analyzer. *Journal of Computational Chemistry* **2012**, *33* (5), 580-592. DOI: 10.1002/jcc.22885.

(17) Lu, T. A comprehensive electron wavefunction analysis toolbox for chemists, Multiwfn. *The Journal of Chemical Physics* **2024**, *161* (8), 082503. DOI: 10.1063/5.0216272.
(18) Lu, T.; Chen, F. Quantitative analysis of molecular surface based on improved Marching Tetrahedra algorithm. *Journal of Molecular Graphics and Modelling* **2012**, *38*, 314-323. DOI: 10.1016/j.jmgm.2012.07.004.
(19) Philippi, F.; Rauber, D.; Eliasen, K. L.; Bouscharain, N.; Niss, K.; Kay, C. W. M.; Welton, T. Pressing matter: why are ionic liquids so viscous? *Chemical Science* **2022**, *13* (9), 2735-2743. DOI: 10.1039/D1SC06857A.
(20) de Souza, B. GOAT: A Global Optimization Algorithm for Molecules and Atomic Clusters. *Angewandte Chemie International Edition* **2025**, *64* (18), e202500393. DOI: 10.1002/anie.202500393.
(21) Bannwarth, C.; Ehlert, S.; Grimme, S. GFN2-xTB—An Accurate and Broadly Parametrized Self-Consistent Tight-Binding Quantum Chemical Method with Multipole Electrostatics and Density-Dependent Dispersion Contributions. *Journal of Chemical Theory and Computation* **2019**, *15* (3), 1652-1671. DOI: 10.1021/acs.jctc.8b01176.
(22) Bannwarth, C.; Caldeweyher, E.; Ehlert, S.; Hansen, A.; Pracht, P.; Seibert, J.; Spicher, S.; Grimme, S. Extended tight-binding quantum chemistry methods. *WIREs Computational Molecular Science* **2021**, *11* (2), e1493. DOI: 10.1002/wcms.1493.
(23) Garcia-Ratés, M.; Neese, F. Effect of the Solute Cavity on the Solvation Energy and its Derivatives within the Framework of the Gaussian Charge Scheme. *Journal of Computational Chemistry* **2020**, *41* (9), 922-939. DOI: 10.1002/jcc.26139.
